# Four Months of a School-Based Exercise Program Improved Aerobic Fitness and Clinical Outcomes in a Low-SES Population of Normal Weight and Overweight/Obese Children With Asthma

**DOI:** 10.3389/fped.2018.00380

**Published:** 2018-12-11

**Authors:** Kim D. Lu, Dan M. Cooper, Fadia Haddad, Shlomit Radom-Aizik

**Affiliations:** Department of Pediatrics, Pediatric Exercise and Genomics Research Center, University of California, Irvine, Irvine, CA, United States

**Keywords:** asthma, obesity, school, low SES, aerobic fitness

## Abstract

**Introduction:** Fitness can improve asthma management. However, children from disadvantaged and minority communities generally engage less in physical activity, and have increased obesity and asthma disease burden. The goal of this pilot study is to evaluate (1) the feasibility of an exercise intervention program in a school-based setting (attendance and fitness improvement) and (2) the effect of the intervention on fitness, asthma, and clinical outcomes in normal weight and overweight/obese children with asthma from low-SES population.

**Materials and Methods:** Nineteen children, ages 6–13 years, from two elementary schools in Santa Ana, CA, a population with high percentage of Hispanic and low socioeconomic status, participated. Training sessions occurred at the schools during afterschool hours (3 sessions weekly × 4 months) and included mainly aerobic age-appropriate activities/games and a small component of muscle strength. Before and after the intervention, evaluations included pulmonary function testing, cardiopulmonary exercise testing (peak $\dot{\text{V}}$O_2_), assessments of habitual physical activity, body composition (DXA), asthma questionnaires, and blood (cardiometabolic risk factors).

**Results:** Seventeen of 19 participants completed the study. Adherence to the program was 85%. Based on BMI %ile, 11 of the participants were overweight/obese and 8 were normal weight. Ten participants had persistent asthma and 9 children had intermittent asthma. Training was effective as peak $\dot{\text{V}}$O_2_ improved significantly (8.1%, SD ± 10.1). There was no significant change in BMI %ile but a significant improvement in lean body mass (1%, SD ± 2.0) and decrease in body fat (1.9%, SD ± 4.6). Asthma quality of life outcomes improved following the intervention in symptoms, emotional function, and overall. There was no change in asthma control or pulmonary function. Five of 10 participants with persistent asthma decreased their maintenance medications. Lipid levels did not change except HDL levels increased (46.1 ± 8.4 mg/dL to 49.5 ± 10.4 mg/dL, *p* = 0.04).

**Discussion:** A school-based exercise intervention program designed specifically for children with asthma for a predominantly economically disadvantaged and minority population was feasible with good adherence to the program and substantial engagement from the schools, families and participants. The exercise intervention was effective with improvement in aerobic fitness, body composition, asthma quality of life, and lipid outcomes, setting the stage for a larger multicenter trial designed to study exercise as an adjunct medicine in children with asthma.

## Introduction

Asthma is the most common chronic disease of childhood, which, despite advances in therapy, continues to cause substantial morbidity among the 6 million children affected in the U.S. (1). Patients with asthma have not been spared by the unrelenting childhood obesity epidemic and it is well established that obesity is a significant risk factor for both asthma development and increased morbidity (2, 3). While the mechanisms linking asthma and obesity are likely multifactorial, poor fitness and physical inactivity may play a role (4, 5).

Studies suggest that better physical fitness can improve asthma symptoms and control in addition to the known benefits for cardiovascular health (6). Exercise intervention programs in children and adults with asthma have been shown to improve aerobic fitness, quality of life, asthma control, airway hyper-responsiveness, and airway inflammation (7). However, studies demonstrating the impact of improved fitness in children of low socioeconomic status (SES) and minority populations, which are disproportionately affected by higher asthma and obesity rates and morbidity, are lacking (8–10). The challenges faced in implementing sustainable lifestyle interventions in these communities include: (1). Accessible and safe venues for physical activity for children is limited and (2). Ensuring robust interventions (for example, training goals of at least 80% of maximum heart rate (HR) and a minimum of 3 sessions per week) to lead to measurable changes in physical fitness (11–13). Without a measure of physical fitness, unfortunately absent in many exercise training studies in children and adults, the impact of the intervention on conditions like asthma is uninterpretable. In a review of exercise training on asthma outcomes, Wanrooij et al concluded that training programs should last at least 3 months with at least 2 sessions per week with a personalized training intensity (at ventilatory threshold or 80% of HR max) (14). School-based intervention studies are appealing for these communities because schools are considered as safe places by parents. Moreover, as data continue to support the beneficial role played by exercise on academic performance (15), schools are increasingly motivated to host and sustain programs designed to enhance physical activity (16). To the best of our knowledge, no exercise intervention studies to date have targeted children with asthma in predominantly lower-SES communities.

The goal of this pilot study was to evaluate (1) the feasibility of an exercise intervention program in a school-based setting, specifically whether we were able to improve fitness in this setting and whether we had sufficient attendance of the training; and (2) the effect of the intervention on fitness, asthma, physical activity and clinical outcomes in a group of normal weight and overweight/obese children with asthma from a predominantly low-SES Hispanic population.

## Methods

### Study Participants

Nineteen children, ages 6–13 years, participated in the study. Inclusion criteria included participants with a current physician diagnosis of asthma (asthma symptoms or medication use in the past 12 months) and no disease or disability that would impair participation in a vigorous exercise program. Participants with asthma exacerbation requiring systemic corticosteroids in the past month, any condition that would preclude exercise, obesity-related complications (i.e., type 2 diabetes), or pregnancy were excluded. This study was approved by the institutional review board at the University of California, Irvine (UCI), and informed consent and assent were obtained from all participants and their legal guardians. All participant expenses were covered by the research program.

### Study Design

We developed relationships with two local elementary schools in Santa Ana, CA, a population with high percentage of Hispanic and low socioeconomic status. The schools contacted all students with diagnosis of asthma or albuterol prescription to attend information sessions regarding the research program. Approximately 30–40 total children/families attended the information sessions, with approximately 25 children eligible to participate (current physician diagnosis of asthma) and 19 children were consented to participate.

The study included two separate visits to the UCI Pediatric Exercise and Genomics Research Center (PERC) Human Performance Laboratory (HPL) before and after a 16-week school-based exercise intervention program. Each visit to the HPL included assessments of fitness and physical activity, body composition, lung function, and asthma questionnaires.

### Anthropometric Measurements

Standard calibrated scales and stadiometers were used to determine height and body mass. Body mass index [BMI = weight/height^2^ (kg/m^2^)] percentile was calculated using the published standards from the Centers for Disease Control (CDC), National Center for Health Statistics, USA (17). Normal weight is defined as BMI 5th to < 85th %ile, overweight is 85th to < 95th %ile, and obese is ≥ 95 %ile.

Dual x-ray absorptiometry (DXA) was used to determine body composition, including lean body mass and percent body fat, using a Hologic QDR 4500 densitometer (Hologic Inc., Bedford, MA, United States). Participants were scanned in light clothing while lying supine. The DXA instrument was calibrated using the procedures provided by the manufacturer, and DXA scans were performed and analyzed using pediatric software. Percent fat categories were calculated based on body fat reference curves for children (18). The 2nd, 85th, and 95th percentiles define the cut-off points for underfat, overfat, and obese.

### Pulmonary Function Testing

Volunteers were requested to hold all medications, including inhaled corticosteroids, for at least 24 h prior to each visit. Spirometry was performed at baseline and 10–15 min following an exercise challenge (ramp test). Spirometry included FVC, FEV_1_, forced expiratory flow, and mid-expiratory phase (FEF_25−75_) measured in triplicate (V_max_229; Sensormedics, Yorba Linda, CA) according to American Thoracic Society (ATS) guidelines (19). Exercise-induced bronchoconstriction (EIB) was present if participants demonstrated ≥ 12% decrease in FEV_1_ following exercise challenge(19). If participants demonstrated EIB, they were given inhalation of albuterol and spirometry was repeated.

### Asthma Questionnaires

Asthma control was assessed using the Childhood Asthma Control Test for participants ≤ 11 years and the Asthma Control Test if > 11years of age (20).

The Children's Health Survey for Asthma (CHSA) is a 48-item validated questionnaire completed by parents with 5 subscales: (1) physical health, (2) child activity, (3) family activity, (4) child emotional health, and (5) family emotional health (21). Primary caregivers were asked, using a 5-point Likert scale, how much of the time their child (or family) experienced each item as a result of asthma during the past 4 weeks. This self-report measure also assesses healthcare utilization, asthma triggers, and family demographics (21).

The Pediatric Asthma Quality of Life questionnaire, which assesses physical, emotional, and social impairment due to asthma over the previous week, was completed as well (22). There are 23 items distributed in three domains: activity limitations, symptoms, and emotional function. All items are similarly answered by means of a 7-point Likert scale, ranging from 1 (severely affected) to 7 (unaffected). The minimally important difference established for this instrument is 0.5 points.

Asthma classification (intermittent or persistent) was based on EPR guidelines (23). Caregivers also reported all current asthma medication prescriptions, which were brought to study visits for confirmation.

### Physical Activity Monitoring

Habitual physical activity and sedentary time were assessed during awake time (8 am to 8 pm) for 7 days using Actigraph GTX3, which was worn on the waist at baseline and end of the study. Twelve-hour daytime activity data were analyzed for weekdays and weekends using Actilife software. Physical activity was classified into sedentary, moderate, and vigorous levels according to the cut points set by Evenson Children 2008 (24). Cut points based on vector magnitude <100 counts per minute (CPM) were scored as sedentary, ≥2,296 CPM were scored as moderate, and ≥4,012 CPM were scored as vigorous physical activity.

### Cardiopulmonary Exercise Test (CPET)

Each subject performed a ramp-type progressive cycle ergometer exercise test using the SensorMedics metabolic system (Vmax 229, Yorba Linda, CA, United States). Following 3 min of sitting comfortably without pedaling (rest) on the cycle ergometer breathing through a mouth piece and 1 min of unloaded pedaling, the work rate (determined by weight and overall assessment of activity level) was incremented at 10–20 W/min to the limit of the participant's tolerance. Participants were vigorously encouraged during the high-intensity phases of the exercise protocol. Gas exchange was measured breath-by-breath and peak $\dot{\text{V}}$O_2_ was determined when RER ≥1.0 (25) and was calculated as the highest 20-s rolling average in the last minute of exercise in absolute values (l/min), relative to body mass (ml/kg/min) and lean body mass (ml/kg/min). Percent predicted peak VO_2_ was determined using published norms (26).

### Blood Sample Collection

Participants arrived to PERC HPL in the morning following > 8-h fast. Blood was collected and sent to the UCI Clinical Pathology Lab for complete blood count (CBC) with differential, complete metabolic panel, lipid panel, and Immunoglobulin E.

### Exercise Training Intervention

Participants completed a 16-week aerobic exercise training intervention (45 min session, 3 days a week) at their local schools during after-school hours. Prior to the beginning of exercise activities, the trainer ensured that the participants had their bronchodilator readily available. Participants were pretreated with bronchodilators prior to exercise activities if specified in their asthma action plan. Training was done in small groups of 4–6 children and included a variety of age-appropriate activities using small equipment (e.g., soccer, basketball and football balls, stability exercise balls, Bosu balls, jump ropes, hula hoops, cones, and scooter boards with safety handles. Training was led by experienced physical education specialists who were assisted by undergraduate student volunteers. Each session included a 5-m warm up, 5-min cool down (at the end), and a combination of aerobic and resistance exercises. Exercises were similar to activities in Physical Education classes. During weeks 1 and 2, aerobic activities (e.g., playing small group games and adapted ball games, basketball, soccer, badminton) were performed for 15 to 20 min. The duration of the aerobic exercises gradually increased up to 45 min by weeks 10 through 16. In addition to the aerobic exercises, participants performed resistance exercises (e.g., band exercises, push-ups, and pull-ups). These exercises were done in sets, starting from low number (4–6) of repetitions within each set, and gradually increased to 10–12 repetitions in a set. The number of sets was also gradually increased based on the participants' strength. Resistance exercises took approximately 20–25 min in the first few weeks and gradually decreased in time in parallel to the increase in time for the aerobic exercises.

To record exercise intensity, participants wore a heart rate (HR) monitor (PolarE600) during the sessions. Number of minutes spent on target HR (bpm) > 145 bmp (70–80% of maximum HR) are reported for sessions that had >40 mins of recording time as means ± standard deviations.

### Statistical Analysis

Data are presented as mean [standard deviation (SD)], median [interquartile range (IQR)], or proportion (*n*, %). The effect of exercise training was assessed using a paired mean-comparison *t*-test or Wilcoxon's signed-rank test as appropriate. All tests were two-tailed and *p* ≤ 0.05 were considered significant. Statistical analysis was performed using Stata version 11 (StataCorp LLC, College Station, TX, United States).

## Results

### Demographics

Nineteen participants, mean age of 9 years and 68% male, participated in the study (Table 1). All participants were Hispanic and 63% with household income < $30,000. Ten of the 19 participants had persistent asthma and 9 participants had intermittent asthma based on NAEPP criteria (23).

**Table 1:** Baseline demographics (*n* = 19).

Age in years, mean, and range	9 (6–13)
Sex, *n* (%)	
Male	13 (68)
Race/Ethnicity, *n* (%)	
Hispanic	19 (100)
Household income^*^, *n* (%)	
<$30,000	12 (63)
$30,000–60,000	2 (11)
>$60,000	3 (16)
BMI Categories, *n* (%)	
Normal weight (5– < 85%ile)	8 (42)
Overweight (85– < 95%ile)	5 (26)
Obese (≥95%ile)	6 (32)
Physical activity levels, median (IQR) in min per 12 h awake	
Sedentary time	515 (496, 549)
Moderate activity	33 (26, 39)
Vigorous activity	28 (17, 37)

* *Median household income in Orange County, CA is $81,837; median household income in Santa Ana, CA is $61,895 (2017 census data)*.

### Training Program

Seventeen of the 19 participants completed the 16-week exercise training intervention. Two participants did not complete the training intervention due to conflicts with other activities and did not complete any post-intervention measurements. There were no adverse events or injuries during the intervention. The average number of sessions attended by participants was 40 sessions with 85% attendance rate. During the training sessions, participants exercised for 29.3 (5.6) mins (average range per participant 18 to 42 mins/session) in the target HR >145 bpm (mean of 79% (8) of HR max).

### Cardiopulmonary Fitness (Table 2)

**Figure 1:**
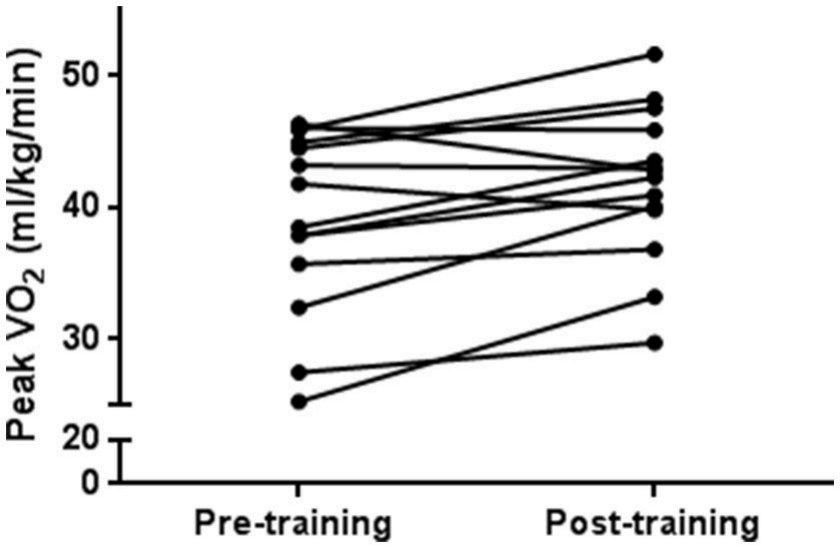
Aerobic fitness improves following exercise training. Each black circle and line pair represents one participant from pre-training to post-training. There was a statistically significant training effect on peak $\dot{\text{V}}$O_2_ (*p* = 0.008).

Mean peak $\dot{\text{V}}$O_2_ was 39.8 ml/kg/min (8.6) and 87.5% predicted (17.8) at baseline. Absolute peak $\dot{\text{V}}$O_2_, peak $\dot{\text{V}}$O_2_ (per body weight) and peak $\dot{\text{V}}$O_2_ (per lean body mass) were all significantly improved [13.3% (22.6), 8.1% (10.1), and 5.2% (12.3), respectively, all *p* < 0.01] from baseline to after the training program (Figure 1 and Table 2).

**Table 2:** Aerobic fitness before and after training.

	**Pre-training mean (SD)**	**Post-training mean (SD)**
Absolute peak $\dot{\text{V}}$O_2_ in L/min	1.5 (0.5)	1.7 (0.5)^*^
Peak $\dot{\text{V}}$O_2_ (per body mass) in ml/kg/min	38.5 (7.1)	41.1 (6.2)^*^
Peak $\dot{\text{V}}$O_2_ (per lean body mass) in ml/kg/min	57.4 (7.3)	60.9 (5.9)^*^

* *p < 0.05 paired t-test for training effect*.

### Body Composition

Forty two percent of participants were normal weight and 58% were overweight/obese. There was a change in weight ranging from 2 kg weight loss to 4.7 kg weight gain, as well as an increase in height ranging from 0 to 6 cm at the end of the training program. There was no significant change in BMI %ile following training.

At baseline, mean % of body fat was 33.8% (6.9) with all participants overfat (≥ 85th to < 95th %ile) or obese (≥ 95th %ile) based on % body fat categories except for one participant in healthy fat category. Following the exercise training program, % of lean body mass increased by an average of 1.1% (2.0), *p* = 0.04, and % of body fat decreased by an average of 1.9% (4.6), *p* = 0.04.

### Asthma Outcomes (Table 3)

#### Healthcare Utilization

There were no hospitalizations or emergency department visits during the study. One participant had a sick visit with their primary care physician before and after training.

**Table 3:** Asthma characteristics.

	**Pre-training**	**Post-training**
**Spirometry, mean (SD)**		
FVC % predicted	107.8 (16.2)	104.3 (13.8)
FEV_1_ % predicted	105.1 (19.1)	101.8 (17.2)
FEV_1_/FVC	84.9 (7.1)	84.4 (9.2)
Exercise-induced bronchoconstriction, n (%)	3 (16)	1 (16)
**Asthma related Quality of Life, median (IQR)**		
Overall	4.5 (4.0, 6.4)	6.0 (4.4, 6.5)^#^
Activity limitations	4.6 (4.1, 6.0)	5.6 (4.3, 6.5)^#^
Symptoms	5.0 (4.0, 6.5)	6.2 (4.9, 6.5)^*^^#^
Emotional Function	4.8 (3.1, 6.4)	6.0 (4.0, 6.7)^#^
**Asthma Control**		
Mean score (SD)	21.2 (3.5)	22.1 (3.7)
Uncontrolled, n (%)	4 (24)	3 (18)
**Children's Health Survey for Asthma, mean (SD)**		
Physical Health	90.0 (12.2)	93.6 (7.2)
Activity (child)	86.8 (20.2)	95.6 (7.7)
Activity (family)	80.4 (15.4)	85.5 (4.4)
Emotion (child)	90.6 (15.7)	94.4 (11.4)
Emotion (family)	68.8 (20.8)	82.5 (14.3)^*^

Asthma control: c-ACT, ACT. Uncontrolled score ≤ 19. Quality of life evaluated by PAQLQ,

# minimally important difference = 0.5,

* *p < 0.05 paired t-test or Wilcoxon's rank sum test*.

#### Asthma Control

Seventy-six percent of participants had well-controlled asthma at baseline (ACT score > 19). There was no significant change in asthma control following exercise training intervention.

#### Asthma Quality of Life

Symptoms, activity limitation, emotional function, and overall score all improved with change in median PAQLQ score of ≥0.5, which is considered a clinically important improvement, following training. Using the Children's Health Survey for Asthma, the emotion (family) domain was significantly improved following training.

#### Medications

Five out of 10 participants with persistent asthma reduced their maintenance medications by the end of the exercise training intervention.

#### Pulmonary Function

There was no significant change in spirometry following the exercise training intervention. Three participants had exercise-induced bronchoconstriction at baseline that was not present after training. One participant had exercise-induced bronchoconstriction after training that was not present at baseline.

#### Physical Activity and Sedentary Time

Participants had a median of 28.6 min (IQR 25.4, 38.3), 30.2 min (IQR 14.7, 35.8) of moderate and vigorous activity time, respectively during weekdays and 37.2 min of moderate (IQR 25.8, 42.0), 26.7 min of vigorous (IQR 20.1, 35.6) activity during weekends at baseline. There was a median of 510.6 min (IQR 488.5, 529.6) of sedentary time during weekdays and 527.1 min (IQR 497.1, 574.6) during weekends at baseline. There was no significant difference in moderate, vigorous activity or sedentary time between normal weight and overweight/obese participants. Overall, there was no significant difference in moderate, vigorous activity time or sedentary time following the intervention. However, there was a trend of increased habitual physical activity time and decreased sedentary time on weekends in normal weight but not overweight/obese children with asthma following training.

#### Lipid Panel (Table 4)

Mean total cholesterol levels were 145 mg/dL (25.4) and one participant with elevated level at baseline (> 200 mg/dL). Mean triglycerides were 79.5 ml/dL (34.5) at baseline and one participant with elevated level at baseline (> 150 mg/dL). Mean LDL levels were 83.1(26.2) at baseline. Mean HDL levels were 46.1 ml/dL (8.4) with two participants with low levels at baseline (< 40 mg/dL). HDL levels improved from a mean of 46.1 to 49.5 mg/dL after training (*p* < 0.04). Participants with abnormal lipid levels were all obese (BMI %ile ≥ 95). Total cholesterol, LDL, and triglycerides levels were not significantly different after training; however, participants with elevated total cholesterol or triglyceride levels at baseline all improved to within normal limits following training.

**Table 4:** Lipid levels before and after exercise training.

	**Pre-training, mean (SD)**	**Post-training, mean (SD)**
**Lipid panel, mg/dL**		
Total cholesterol	145.1 (25.4)	148.9 (21.5)
Triglycerides	79.5 (34.5)	78.6 (27.5)
HDL	46.1 (8.4)	49.5 (10.4)^*^
LDL	83.1 (26.2)	83.7 (20.2)

* *p < 0.05 paired t-test*.

## Discussion

This pilot study is the first to test a school-based program specifically designed to improve aerobic fitness in low-SES children with asthma. We found evidence that the 16-week aerobic exercise program was feasible in that there was a high attendance rate among participants for the training sessions and all but two participants completed the training. In discussions with families, the majority of participants enjoyed their involvement in the program, and parents/guardians were supportive of their children's participation. School personnel communicated to our team that they would welcome studies of this nature in the future. A major strength of the pilot was our ability to demonstrate improvements in cardiorespiratory fitness in a school-based setting (27). We showed significant increases in robust physiological metrics including $\dot{\text{V}}$O_2_max, lean body mass, and an increase in HDL. Finding these measurable effects of the training program addresses a key deficiency in many previous exercise training studies in children because the exercise-training associated biological improvements can be used to gauge the impact of the training on disease related phenotypes, in this case, asthma-related metrics.

Previous lifestyle interventions have required participants to travel to centralized training facilities. This approach is not practical or sustainable in communities with high risk populations, particularly in lower-SES and minority areas. In contrast, school-based interventions, considered by parents and children as familiar, safe, and accessible venues, have been shown to improve cardiorespiratory fitness and physical activity in children by utilizing exercise sessions in addition to physical education classes (28, 29). Our project was possible only because of the strong relationships built with local schools and their active engagement with the families. In the communities where we partnered with the schools, the school grounds were among the only areas accessible and safe for the participants to engage in active play. We partnered with the after-school activities to coordinate space and time. While sessions were led by physical education specialists, these activities could be planned and executed by any trained staff. Utilizing HR monitors allowed the participants to get into the target zone. We found that utilizing array of small equipment and games (felt by the participants to be fun) from session to session was important to keep the children engaged throughout the length of the intervention.

Obesity and overweight status contribute to a wide range of health threats in children, ranging from increased cardiovascular disease risk to type 2 diabetes (30, 31). At baseline, 42% of our participants were normal weight using BMI %iles; however, when looking at body composition using % body fat (a metric obtained by DXA, not possible with BMI alone), all but one were equal to or greater than the 85th percentiles, highlighting the limitations of BMI in defining obesity in children during the period of growth and development in which rapid changes in body composition occur. Furthermore, BMI cannot distinguish between fat mass and lean mass and may fail to identify as many as 25% of children with excess body fat (32). Despite this, many health interventions in children target body composition (usually measured solely as BMI), most likely due to the relative ease of measuring height and weight and the additional expense and time required for assessments like DXA. These interventions are typically multifaceted, incorporating diet, physical activity, and motivational counseling. While the rationale for these complex interventions is understandable, multifaceted designs confound the investigators' ability to identify which component of the intervention (e.g., diet or exercise) led to the improved health outcome.

There are only a few studies targeting weight loss as a means to ameliorate asthma outcomes in obese asthmatic children (33–36). The interventions used varied from multifactorial interventions with both exercise and nutritional counseling to diet alone. Overall, weight loss was associated with improvements in asthma control, asthma-related quality of life, and exercise-induced bronchoconstriction (33, 37, 38). Jensen et al was the only pediatric study to measure body composition (using DXA) and found correlations between changes in % body fat or % lean mass and inflammatory markers (for example, C-reactive protein, exhaled nitric oxide). Lucas et al retrospectively compared healthy and asthmatic children in a 12-week nutrition and physical activity intervention in children who were overweight/obese or at risk of obesity and found significant improvements in BMI percentile or z-score as well as $\dot{\text{V}}$O_2_max (39). Fifty-eight percent of the sample completed the program though there was no difference in attrition between healthy and asthmatic children. However, the study did not report any asthma outcomes. None of the previous studies focused solely on improvements in aerobic fitness or evaluated relationships between fitness and asthma outcomes. In our study, which primarily focused on aerobic fitness, we did not observe significant improvement in BMI %ile, but we did find improvements in lean body mass. The anti-inflammatory effect of routine physical activity and exercise may be mediated through several mechanism(s), including a reduction in NF-κβ levels, glucocorticoid receptor expression, or anti-inflammatory cytokines (40, 41). Other possible mechanisms of exercise training on asthma include increasing the ventilatory threshold, reduction in the perception of breathlessness, or improving exercise tolerance (42–44). Improving fitness may mitigate chronic inflammation, which is one of the key common features of both obesity and asthma (45), and reducing chronic inflammation may lead to improvements in asthma outcomes and overall health.

We found improvements in asthma quality of life in symptoms, emotional function, and overall score using the PAQLQ following the exercise program. We also found improvement in the emotion (family) domain using the Children's Health Survey for asthma. In contrast to the other intervention studies targeting obese asthmatic children, we did not find any significant difference in asthma control or pulmonary function following the exercise intervention (37). The majority of our study participants had well-controlled asthma as well as normal lung function at baseline. Additionally, half of the participants with persistent asthma were able to decrease or discontinue their daily medications which is consistent with other studies reporting a reduction in inhaled or systemic corticosteroids following an exercise training intervention (42, 46).

The exercise program was effective in improving aerobic fitness with no significant difference in habitual physical activity or sedentary behavior. Previous studies evaluating the relationship between CPET measures of physical fitness and habitual physical activity have consistently shown relatively weak correlations between these two variables (12, 47), dispelling the fear that an organized exercise intervention would reduce habitual physical activity. Sedentary behavior appears to be an independent risk factor for worse cardiometabolic health and obesity (48).

## Study Limitations

Our project was targeted at a predominantly Hispanic, lower SES community in Southern California and our findings might not be generalizable to all children with asthma. However, we believe that there is a commonality in the willingness of all children, regardless of race or ethnicity to participate in fun exercise activities. Additionally, this was a pilot feasibility project with a small sample size and we cannot assess the effectiveness of our results in comparison to a control group that was not exposed to the exercise-training intervention.

## Conclusion

A school-based exercise training intervention is a promising approach to promote fitness and health outcomes in children with asthma, including those from lower SES minority communities. Utilizing the schools provides a safe venue for children and serves as a potentially sustainable model for fitness interventions in children. Our study sets thestage for larger multicenter randomized controlled trials designed to improve fitness and study how exercise can be best used as an adjunct therapy to improve asthma outcomes.

## Ethics Statement

This study was carried out in accordance with the recommendations of University of California Institutional Review Board Committee with written informed assent and consent from all subjects and their parents/guardians. All subjects and their parents/guardians gave written informed assent/consent in accordance with the Declaration of Helsinki. The protocol was approved by the University of California Institutional Review Board Committee.

## Author Contributions

KL and SR-A: Conception and design of the research study; KL, FH, DC, and SR-A: Data analysis and interpretation; KL, FH, DC, and SR-A: Review and approval of manuscript.

### Conflict of Interest Statement

The authors declare that the research was conducted in the absence of any commercial or financial relationships that could be construed as a potential conflict of interest.
